# Serum levels of gamma-glutamyl transpeptidase in relation to HCC human biology and prognosis

**DOI:** 10.15761/jts.1000446

**Published:** 2020-09-17

**Authors:** BI Carr, H Akkiz, HG Bag, U Karaoğullarından, K Yalçın, N Ekin, A Özakyol, E Altıntaş, HY Balaban, H Şimşek, A Uyanıkoğlu, A Balkan, S Kuran, O Üsküdar, Y Ülger, B Güney, A Delik

**Affiliations:** 1 İnönü University, Malatya, Turkey; 2 Çukurova University, Adana, Turkey; 3 Dikle University, Diyarbakır, Turkey; 4 Eskişehir Osmangazi University, Eskişehir, Turkey; 5 Mersin University, Mersin, Turkey; 6 Hacettepe University, Ankara, Turkey; 7 Harran University, Şanlıurfa, Turkey; 8 Gaziantep University, Gazientep, Turkey

**Keywords:** HCC, survival, GGT

## Abstract

**Background and aim:**

Hepatocellular carcinoma (HCC) biomarkers are limited, as even the best studied, alpha-fetoprotein (AFP), is elevated in no more than 50% of HCC patients. The aim was to evaluate several serum liver function tests in relation to survival and tumor characteristics in a large cohort of Turkish HCC patients.

**Methods:**

We retrospectively examined the serum levels of gamma glutamyl transpeptidase (GGT) in relation to patient survival.

**Results:**

Kaplan-Meier analysis showed that only GGT and albumin amongst liver function tests, were significantly associated with survival. Survival worsened with increase in GGT levels semi-quantitatively. Increase in GGT levels was also found to significantly correlate with an increase in maximum tumor diameter from 4.5 to 7 cm, a 20-fold increase in serum alpha-fetoprotein level, an increase in tumor multifocality from 20 to 54% of patients, and a doubling in percent of patients with portal vein thrombosis (PVT) from 20 to 40%. Serum GGT levels also showed significant survival differences for patients with low AFP levels. A doublet combination of serum GGT with albumin levels was associated with higher hazard ratios in a Cox regression analysis, as compared with single parameter GGT. The combination parameter pair was also prognostically useful in the low-AFP patient subcohort and was associated with significant differences in patient tumor characteristics.

**Conclusions:**

Serum GGT levels and especially combination serum GGT plus albumin levels, were significantly associated both with HCC patient survival and tumor aggressiveness characteristics, regardless of AFP levels in a large Turkish cohort. This might be especially useful since the majority of HCC patients do not have elevated levels of AFP.

## Introduction

Hepatocellular carcinoma (HCC) prognosis has in general been shown to depend on 2 separate yet related considerations, namely tumor and non-tumor characteristics. The former includes tumor size and multiplicity, presence or absence of portal vein invasion by tumor (PVT), degree of tumor differentiation, serum levels of serum alpha-fetoprotein [1–4] and an immature form of prothrombin called desgamma carboxy prothrombin or DCP [5], which in turn is associated with increased incidence of PVT [6]. The latter includes inflammation and other damage to the underlying liver, as reflected in standard clinical liver function tests [7,8] and more recently in parameters of systemic inflammation, including platelet-lymphocyte ratio, neutrophil-lymphocyte ratio, C-reactive protein [9,10] and albumin [11] as well as many novel biomarkers and their combinations that are currently under study [12–14].

Amongst the standard liver function tests, gamma-glutamyl transpeptidase (GGT) stands out as being associated experimentally with pre-neoplastic HCC lesions [15,16]. GGT, also known as gammaglutamyl transferase, has been thought to play a role in HCC growth and development and in resistance to drug toxicity [16,17], being a cell surface enzyme that is involved in glutathione metabolism and is thus important in the maintenance of cellular cysteine levels. Furthermore, GGT has also been considered to be a clinically important prognostic factor [18–21].

In the current work, the significance of serum levels of GGT alone or in combination with other liver function parameters, especially albumin, has been examined in relation to HCC survival and to clinical tumor characteristics. GGT was found to be a useful biomarker for prognosis and for tumor aggressiveness parameters, including in patients with small tumors or in HCC patients who have low serum AFP levels.

## Methods

### Patient data:

We retrospectively analyzed a database of 470 prospectively-accrued non-transplant HCC patients who had both survival data and baseline tumor parameter data, including CT scan information on HCC maximum tumor diameter (MTD), number of tumor nodules and presence or absence of macroscopic portal vein thrombosis (PVT), as well as serum alpha-fetoprotein (AFP) levels; complete blood count; routine serum liver function tests, (total bilirubin, GGTP, ALKP, albumin, transaminases, and patient demographics. Diagnosis was made either via tumor biopsy or according to international guidelines. Database management conformed to legislation on privacy and this study conforms to the ethical guidelines of the Declaration of Helsinki and approval for this retrospective study on de-identified HCC patients was obtained by the Institutional Review Board of each participating institution, as previously reported [22].

### Statistical analysis

Continuous variables were summarized by median, minimum and maximum values. Comparisons between two groups were performed by Mann Whitney U test. For more than two groups, Kruskal-Wallis test and Conover post-hoc method was used. Categorical variables were expressed as count and percentage, comparisons according to these variables were made by Pearson’s chi-square, continuity corrected chi-square or Fisher’s exact test where appropriate. Kaplan-Meier method and Log-Rank test were used for survival analysis. Cox regression was used for Hazard Ratio (HR) estimations. In all analysis two-tailed significance level was considered as 0.05. IBM SPSS Statistics for Windows version 22.0 (NY, USA) was used for statistical analysis.

## Results

### Kaplan-Meier survival analysis for the total cohort

A Kaplan-Meier Survival Analysis for the total HCC cohort was performed, using the clinical tumor characteristics and laboratory liver function characteristics (Table 1). Significance was found for all 4 clinical tumor characteristics of maximum tumor diameter (MTD), tumor number, macroscopic portal vein thrombosis (PVT) and serum alpha-fetoprotein (AFP) levels, as expected. For the remaining blood parameters, total bilirubin levels, alkaline phosphatase (ALKP) levels and platelet counts were not found to be significant, but serum levels of liver function parameters gamma glutamyl transpeptidase (GGT), albumin and aspartate aminotransferase (AST) were found to be significant for survival.

### Serum GGT levels in relation to survival and tumor characteristics

**Figure 1.**
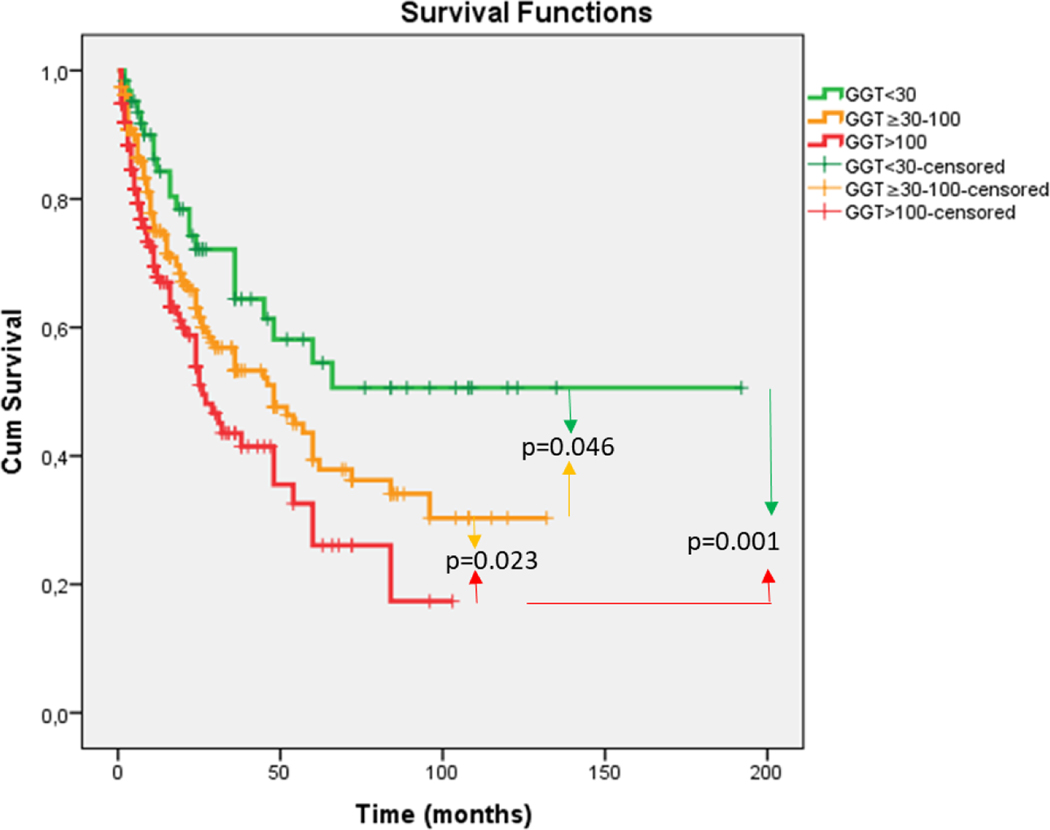
Cumulative survival (mo) as a function of serum GGT levels

Serum GGT levels for the total cohort were trichotomized, and the 3 resulting terciles were examined for their relationship to survival (Tables 2) (Figure 1). Mean survival was found to be 111.18 months (mo.) for GGT levels <30 IU/mL, 61.72 mo. for GGT levels of 30–100 IU/mL and 41.72 mo. for GGT levels >100 IU/ml, p=0.001. The tumor characteristics associated with these 3 GGT terciles were then examined. Statistically significant increases in MTD, AFP levels, tumor number and percent of patients with macroscopic PVT were found with each increase in GGT level (Table 3). GGT levels were then examined in relation to the smallest diameter tumors in the cohort (Table 4). GGT levels increased with each MTD cohort and significant increases were found for tumors <5 cm compared to 5–10 cm; however, levels for <2 cm versus >2–5 cm were not significantly different, both for absolute serum GGT values and for percent of patients with serum GGT >100 IU/mL.

The serum GGT terciles were then examined in patients with low serum AFP <100 IU/mL levels. The results also showed significantly longer (3-fold) survival for patients with low GGT levels <30 IU/mL as compared with highest GGT levels >100 IU/mL (Table 5).

In the AFP<100 IU/mL group, GGTP terciles showed differences in the tumor characteristics, with significantly more tumor multifocality and percent of patients with PVT in the high CRP group compared to the low CRP group (Table 6).

### Parameter doublet combinations with GGT

Combinations of serum GGT alone or with either serum albumin or ALKP were then examined separately for smaller <5 cm and larger >5 cm tumors. For the combinations of GGT with albumin, significant differences in survival were found for high or low levels, regardless of whether patients with smaller (<5 cm MTD) or larger (>5 cm MTD) tumors were examined (Table 7). The HRs for the combinations of GGT with albumin were greater than for GGT alone. Addition of ALKP to GGT however, resulted in significant survival differences only for patients with larger tumors.

### Serum levels of GGT plus albumin together

The clinical correlates of the combination of serum GGT plus albumin levels together, were then examined (Table 8). Normal/low combination levels (GGT <100 IU/mL plus albumin >3.5 g/dL) were compared to abnormal/high combination levels (GGT >100 IU/mL and albumin <3.5 g/dL). Table 8 shows that almost every blood clinical parameter was different, when normal versus abnormal combination parameters were compared, except for platelets and LDL. Especially notable was the greater that 6-fold difference in CRP levels between the 2 groups. Similarly, when the tumor characteristics between the 2 groups were compared, every tumor parameter (MTD, AFP, tumor number, percent patients with PVT) were significantly worse in the abnormal/high combination parameter GGT plus albumin pair, compared to the low/normal GGT plus albumin combination values, all p<0.001 (Table 9 ). These tumor parameter differences were also found to all be significant for patients with large >5 cm tumors (Table 9); but for patients with small <5 cm tumors, none of the tumor parameters were significantly different (Table 9).

## Discussion

The results reported here are of newly available survival data from a recently formed Turkish multi-institutional HCC collaborative group [22]. Kaplan-Meier analysis showed 4 out of 6 significant liver factors for survival, including GGT, AST, albumin and AST, but not bilirubin or ALKP. AFP was significant but was not included in further analysis or model-building, as less than 60% of HCC patients have elevated levels [23–25]. Focus was initially made on GGT, due to previous reports on its importance as a biomarker in experimental hepatocarcinogenesis, as well as several reports of its potential as a clinical HCC prognostic biomarker [15–21]. We found that increasing serum GGT levels were not only significantly associated with decreasing survival, but also with significantly worse levels of the 4 tumor parameters-MTD, tumor multifocality, serum AFP levels and percent of patients with macroscopic PVT. For the mean survival times, each serum GGT level was significantly different from each other serum GGT level. Patients with different MTD size bands were further examined for GGT levels and percent with elevated GGT, to determine if GGT might be useful for the search for very small tumors. Although there was an increase in serum GGT levels for each size band, we could not discriminate between patients in the <5 cm MTD bands. The same approach was taken to examine the important low AFP group of patients in relation to serum GGT levels. The 2 GGT groups showed a 3-fold survival difference in the total cohort and in the groups with different serum GGT levels. The 4 tumor characteristics were all worse for the shorter survival, highest serum GGT level group as compared with the longer survival, lowest serum GGT level group, especially with multifocality and percent of patients with PVT being significantly different in the 2 GGT groups.

Serum GGT levels were also combined with serum albumin levels to form parameter doublets. The hazard ratios (HRs) of the doublets were higher than for singlet GGT alone, when patients with either smaller or larger size tumors were considered separately. However, addition of ALKP to GGT did not improve on GGT-related survival for smaller tumors. Combination serum liver parameters were then examined, using doublet GGT plus albumin, and survivals for patients with normal/low doublet combination parameters were found to be significantly higher (2- fold) than for patients with abnormal doublet combination values, whether patients with larger or with smaller tumors were considered and this combination of parameters was better (higher HR) for predicting survival than GGT levels alone. When the 2 combination groups were compared for tumor characteristics, the patients in the worse survival group had significantly worse values for all 4 tumor parameters than for the better survival group.

GGT has been thought to be an HCC biomarker for many years [15,16,18–21], even though it has not been in general clinical practice or decision-making paradigms. However, there is a recent increase in its consideration as a practical marker [19–21], especially for that group of almost 50% of HCC patients who do not have elevated AFP [22–25]. Furthermore, there have been several other reports of the usefulness of GGT in combination with other markers [26–31], as well as a meta-analysis [32]. Of particular note in our analysis, was the usefulness of combination GGT plus albumin in the low-AFP cohort, for whom there are few other validated tumor markers (Tables 11 and 12). There was a significant, 3-fold difference in survival between these 2 groups of low-AFP patients, as well as significant differences in tumor characteristics. Thus, the prognostic usefulness of combination GGT plus albumin are confirmed in this study, in addition to the findings in Chinese HCC patients [26,27].

What might be the functions of HCC in either tumorigenesis or in conjunction with other factors involved in its usefulness in prognosis? GGT has been considered as a biomarker for liver damage and alcohol abuse [33] and is likely associated with hepatic inflammation [30,34]. It is a membrane-bound enzyme involved in the metabolism of glutathione by transferring γ-glutamyl groups and glutathione is a cellular thiol-antioxidant, which can protect cells from oxidant damage by neutralizing reactive oxygen species and free radicals [16,17]. Thus, cellular GGT levels increase under oxidative stress. Conversely, GGT has also been reported to be both a mediator of oxidative cell damage via an increase of reactive oxygen species [35,36], which in turn induce inflammation and contribute to hepatocarcinogenesis, as well as being involved in resistance to cell cytotoxicity by increasing cellular cysteinyl-glycine levels [16,17,37,38]. Precisely how GGT might contribute to HCC growth and invasiveness (PVT) has not been clearly explained. One possible mechanism is through its mediation of free-radical associated mutagenesis [35]. Other possible means are the involvement of the GGT pathway in cell-cycle arrest mechanisms [39] or through metabolomics re-wiring [40]. In addition, GGT deficiency has been shown to be associated experimentally with growth retardation, offering a possible set of mechanisms for an association of over-expression and growth promotion [41,42]. It is also an onco-fetal protein [43,44], similar in this respect to AFP. Finally, an HCC-specific GGT isozyme has been the subject of multiple reports [45–48]. Its function could be consistent with tumor cell growth or metabolism or drug resistance; in fact, they might all be linked.

## Conclusion

This report therefore extends previous observations in a large non-surgical series, by showing that GGT is prognostically useful even for small size tumors and in HCC patients who do not have elevated serum AFP levels and in a description of their associated tumor characteristics. The addition of serum albumin to GGT values slightly enhanced its usefulness. However, GGT also has limitations for discriminating amongst small tumors.

## Figures and Tables

**Table 1.** Kaplan-Meier Survival Analysis for total HCC cohort (n=470)

		Survival time		
		Mean±SE	Median±SE	P
Overall		66.25±4.29	31±3.03	
MTD (cm)	<3	53.00±6.47^a^	36±8.22	
	3–5	54.69±5.50^a^	30±6.89	0.023
	>5	40.87±3.54^b^	24±3.10	
PVT	No	52.92±3.40	31±3.13	<0.001
	Yes	26.24±3.88	9±1.48	
AFP, IU/mL	<100	76.89±5.91^a^	46±7.59	<0.001
	100–1000	41.20±5.11^b^	23±3.60	
	>1000	39.20±5.29^b^	24±5.55	
Multifocality	1	55.00±3.66	31±5.46	0.004
	>1	39.85±4.10	24±4.24	
3 Platelets,10^3^/μL	≤125	53.82±4.27	31±4.14	0.288
	>125	63.25±6.13	29±3.59	
Albumin,g/dL	≤3,5	48.85±3.37	26±2.99	<0.001
	>3,5	83.57±8.01	48±6.16	
T.BIL (mg/dL)	≤1,5	82.18±6.81	48±7.13	0.109
	>1,5	55.46±5.50	29±5.07	
ALKP, IU/mL	≤100	61.36±5.17	45±8.53	0.511
	>100	80.81±7.49	48±8.13	
GGT, IU/mL	≤100	88.52±6.64	48±7.13	0.002
	>100	41.72±4.20	26±3.45	
AST, IU/mL	≤40	102.44±10.36	84±17.93	<0.001
	>40	53.15±3.97	28±3.28	

**Abbreviations:** GGT: Gamma glutamyl transpeptidase (IU/mL); ALB: Albumin (g/dL); AST: Aspartate aminotransferase (IU/L); ALKP: Alkaline phosphatase (IU/mL); T. BIL: Total bilirubin (mg/dL); AFP: Alpha-fetoprotein (IU/mL); MTD: Maximum tumor diameter; PVT: Macroscopic portal vein thrombosis

**Table 2.** Serum GGT levels in relation to survival (For total cohort; all differences are statistically significant.

		Survival time (mo)		Log-rank p-value	Univariate Cox Regression HR (95%C.I.)	HR p-value
		Mean±SE	Median±SE			
Total cohort	GGT<30 IU/mL	111.18±13.04^a^	NA	0.001	reference	
	GGT≥30–100 IU/mL	61.72±4.71^b^	48±8.80		1.59 (0.99–2.52)	0.052
	GGT>100 IU/mL	41.72±4.20^c^	26±3.45		2.22 (1.38–3.56)	0.001

Abbreviation: GGT, gamma glutamyl transpeptidase, (IU/mL)

**Table 3.** Tumor characteristics as a function of serum GGT levels

	GGT<30 IU/mL	GGT≥30–100 IU/mL	GGT>100 IU/mL	
	Median (min.-max.)	Median (min.-max.)	Median (min.-max.)	p
MTD size	4.5 (1–16)	5 (1–24)	7 (0.8–20.5)	<0.001
AFP	9.3 (1.2–12768)	37.1 (0.5–918965)	207.8 (0.5–600000)	<0.001
	%	%	%	p
Tumor # ≤ 2	80.0	62.7	45.6	<0.001
Tumor # > 2	20.0	37.3	54.4	
PVT (−)	78.8	75.9	59.6	<0.001
PVT (+)	21.2	24.1	40.4	

Abbreviations: GGT: Gamma glutamyl transpeptidase: (IU/mL); MTD: Maximum tumor diameter; AFP: Alpha-fetoprotein; PVT: Macroscopic portal vein thrombosis

**Table 4.** Smallest tumor size with elevated serum GGT. Superscript^a^ groups are not different from each other, but are significantlydifferent to superscript^b^ groups

MTD size	Serum GGT values, IU/mL Mean (median;min-max)	% GGT >100 IU/mL
≤2 cm	111.25 (58.5;13–520)^a^	36.4^a^
>2–5 cm	121.71 (80;11–1184)^a^	38.7^a^
>5–10 cm	174.11 (103;12–1058)^b^	52.4^b^
p-value	<0.001	0.021

**Table 5.** Kaplan-Meier Survival Analysis for low AFP (<100 IU/mL) patients Superscript^a^ groups are not different from each other but are significantly different to superscript^b^ groups

		Survival time		
		Mean±SE	Median±SE	p
Overall		76.89±65.31	46±7.59	
MTD (cm)	<5	60.59±5.73	38±12.58	0.202
	≥5	46.40±4.41	35±5.61	
	<30	123.15±15.48^a^	NA	
GGT, IU/mL	30–100	61.44±5.21^b^	57±10.82	0.014
	>100	41.61±4.74^b^	26±7.84	

Abbreviations: GGT: Gamma glutamyl transpeptidase: (IU/mL); MTD: Maximum tumor diameter

**Table 6.** Tumor characteristics as a function of serum GGT levels, within low AFP (<100 IU/mL)

	GGT<30 IU/mL	GGT≥30–100 IU/mL	GGT>100 IU/mL	
	Median (min.-max.)	Median (min.-max.)	Median (min.-max.)	p
MTD size	5 (1–16)	5 (1–24)	6 (0.8–20.5)	0.051
AFP	4.5 (1.2–98.1)^a^	10 (0.5–99)^b^	9.1 (0.5–95.9)^b^	0.010
	%	%	%	p
Tumor # ≤ 2	78.4	68.1	51.5	0.005
Tumor # > 2	21.6	31.9	48.5	
PVT (−)	83.3	83.8	67.0	0.009
PVT (+)	16.7	16.2	33.0	

Superscript

a groups are not different from each other, but are significantly different to superscript

b groups. Abbreviations: GGT: Gamma glutamyl transpeptidase: (IU/mL); MTD: Maximum tumor diameter; AFP: Alpha-fetoprotein; PVT: Macroscopic portal vein thrombosis

**Table 7.** Univariate Kaplan-Meier and Cox regression analysis for duplicate parameters

		Survival time		Log-rank p-value	Univariate Cox Regression HR (95%C.I.)	HR p-value
		Mean±SE	Median±SE			
MTD<5 cm	GGT ≤100	69.67±5.74	60±19.01	0.023	reference	
	GGT >100	40.23±5.82	38±12.15		1.73 (1.07–2.81)	0.026
MTD≥5 cm	GGT ≤100	54.29±5.85	36±8.03	0.011	reference	
	GGT >100	26.01±3.34	24±4.93		1.63 (1.11–2.39)	0.013
MTD<5 cm	GGT ≤100 & ALB>3.5	83.03±8.42	NA	0.002	reference	
	GGT >100 & ALB≤3.5	40.74±7.58	38±10.24		2.99 (1.45–6.19)	0.003
MTD≥5 cm	GGT ≤100 & ALB>3.5	63.10±7.95	45±8.50	<0.001	reference	
	GGT >100 & ALB≤3.5	28.12±3.93	24±5.82		2.67 (1.52–4.71)	0.001
MTD<5 cm	GGT ≤100 & AST≤40	86.29±8.53	NA	0.001	reference	
	GGT >100 & AST>40	37.68±5.95	38±9.21		3.01 (1.47–6.14)	0.002
MTD≥5 cm	GGT ≤100 & AST≤40	53.94±8.33	52±12.19	0.005	reference	
	GGT >100 & AST>40	21.10±3.31	16±3.45		2.25 (1.25–4.07)	0.007
MTD<5 cm	GGT ≤100 & ALKP>100	73.80±9.86	NA	0.346	reference	
	GGT >100 & ALKP≤100	36.66±7.93	26±11.72		1.57 (0.61–4.05)	0.352
MTD≥5 cm	GGT ≤100 & ALKP>100	56.33±8.21	30±16.02	0.037	reference	
	GGT >100 & ALKP≤100	13.11±5.29	9±5.00		2.66 (1.01–7.02)	0.048

Abbreviations: MTD: Maximum tumor diameter; GGT: Gamma Glutamyl transpeptidase (IU/mL); ALB: Albumin (g/dL); AST: Aspartate aminotransferase (IU/L); ALKP: Alkaline phosphatase (IU/Ml)

**Table 8.** Lab values in patients with high or low serum GGTP and Albumin levels in total cohort

	GGTP≤100 & ALB>3.5 & AST≤40	GGTP>100 & ALB≤3.5 & AST>40	
	Median (min.-max.)	Median (min.-max.)	p
Hb, g/dL	14.2(9.3–17.6)	11.9(5.7–17.1)	<0.001
3 Plat, 10^3^/μL	163.5(35–387)	166.5(13–2700000)	0.757
Albumin, g/dL	3.8(3.53–4.88)	2.6(1.2–3.5)	<0.001
CRP, md/dL	1(0.168–162.57)	6(0.13–230.07)	<0.001
ALKP, IU/mL	79(36–335)	202.5(46–1103)	<0.001
GGT, IU/mL	32(12–90)	211(101–1286)	<0.001
AST, IU/mL	29(13–39)	108(41–2061)	<0.001
T.BİL, mg/dL	0.835(0.22–2.1)	1.9(0.3–33.99)	<0.001
Cholesterol, mg/dl	1.14(0.9–2.29)	1.27(0.85–6.06)	<0.001
ALT, IU/mL	23(7–64)	62(11–942)	<0.001
HDL, mg/dL	43(16–96)	27.55(5–89)	<0.001
LDL, mg/dL	105(40.6–237)	97.5(26–310)	0.305

GGT: Gamma glutamyl transpeptidase: (IU/mL); ALB: Albümin (g/dL); AST: Aspartate aminotransferase (IU/L); Plat: Platelets. Other parameter abbreviations on front page.

**Table 9.** Tumor characteristics in patients with high or low serum GGTP and Albumin levels

	GGTP≤100 & ALB>3.5 & AST≤40	GGTP>100 & ALB≤3.5 & AST>40	
	Median (min.-max.)	Median (min.-max.)	p
MTD size	4.8(1.3–16)	7(0.8–20.5)	0.003
AFP	11.2(1.8–51287)	236.5(1–600000)	<0.001
	%	%	
Tumor # ≤2	69.8	43.6	0.004
Tumor # >2	30.2	56.4	
PVT (−)	86.0	58.9	<0.001
PVT (+)	14.0	41.1	

Abbreviations: MTD: Maximum tumor diameter (cm); AFP: Alpha-fetoprotein (IU/mL); PVT: Macroscopic portal vein thrombosis; GGT: Gamma glutamyl transpeptidase: (IU/mL); ALB: Albümin (g/dL); AST: Aspartate aminotransferase (IU/L)

## Abbreviations:

HCC: Hepatocellular carcinoma
PVT: Portal vein thrombosis
AFP: Alpha-fetoprotein
GGTP: Gamma glutamyl transpeptidase
ALKP: Alkaline phosphatase
AST: Aspartate aminotransferase
ALT: Alanine aminotransferase
Alb: Albumin
CRP: C-reactive protein
ESR: Erythrocyte sedimentation rate
Hb: Hemoglobin
Plt: Platelets
T. bili: Total bilirubin
HDL: High density lipoprotein cholesterol
LDL: Low density lipoprotein cholesterol
MTD: Maximum tumor diameter
CT: Computerized axial tomography
MRI: Magnetic resonance imaging
